# Circular functional analysis of OCT data for precise identification of structural phenotypes in the eye

**DOI:** 10.1038/s41598-021-02025-4

**Published:** 2021-12-02

**Authors:** Md. Hasnat Ali, Brian Wainwright, Alexander Petersen, Ganesh B. Jonnadula, Meghana Desai, Harsha L. Rao, M. B. Srinivas, S. Rao Jammalamadaka, Sirisha Senthil, Saumyadipta Pyne

**Affiliations:** 1 L. V. Prasad Eye Institute, Hyderabad, Telangana India; 2 EEE Department, BITS Pilani, Hyderabad Campus, Hyderabad, Telangana India; 3 Department of Statistics and Applied Probability, University of California, Santa Barbara, CA USA; 4 Department of Statistics, Brigham Young University, Provo, UT USA; 5 Health Analytics Network, Pittsburgh, PA USA; 6 Narayana Nethralaya, Bengaluru, Karnataka India; 7 University Eye Clinic Maastricht, Maastricht University Medical Center, Maastricht, the Netherlands; 8 Public Health Dynamics Laboratory, and Department of Biostatistics, Graduate School of Public Health, University of Pittsburgh, Pittsburgh, PA USA

**Keywords:** Diseases, Computational biology and bioinformatics

## Abstract

Progressive optic neuropathies such as glaucoma are major causes of blindness globally. Multiple sources of subjectivity and analytical challenges are often encountered by clinicians in the process of early diagnosis and clinical management of these diseases. In glaucoma, the structural damage is often characterized by neuroretinal rim (NRR) thinning of the optic nerve head, and other clinical parameters. Baseline structural heterogeneity in the eyes can play a key role in the progression of optic neuropathies, and present challenges to clinical decision-making. We generated a dataset of Optical Coherence Tomography (OCT) based high-resolution circular measurements on NRR phenotypes, along with other clinical covariates, of 3973 healthy eyes as part of an established clinical cohort of Asian Indian participants. We introduced CIFU, a new computational pipeline for CIrcular FUnctional data modeling and analysis. We demonstrated CIFU by unsupervised circular functional clustering of the OCT NRR data, followed by meta-clustering to characterize the clusters using clinical covariates, and presented a circular visualization of the results. Upon stratification by age, we identified a healthy NRR phenotype cluster in the age group 40–49 years with predictive potential for glaucoma. Our dataset also addresses the disparity of representation of this particular population in normative OCT databases.

## Introduction

Progressive optic neuropathies such as glaucoma can cause irreversible blindness, especially when left untreated or diagnosed late. Indeed, early detection and management hold the key to slowing the progressive loss of vision and preventing blindness due to many chronic and age-related degenerative eye diseases. Glaucoma, for instance, is the second-leading cause of blindness worldwide^1^. In 2020, an estimated 80 million individuals worldwide had glaucoma and this number is expected to increase to over 111 million by 2040^2^.

There are multiple sources of subjectivity and analytical challenges that are often encountered by the clinicians in the process of early diagnosis and clinical management of degenerative eye diseases. In glaucoma, the functional damage is established most commonly by the occurrence of visual field (VF) loss whereas the structural damage is often characterized by neuroretinal rim (NRR) thinning of the optic nerve head (ONH), and loss of retinal nerve fibers, which are the axons of retinal ganglion cells (RGC). Such thinning could be measured in terms of reduction in either NRR area or NRR thickness. On the functional side, while standard automated perimetry (SAP) has been the gold standard for detection of VF loss, often 30% of RGC loss may have already occurred before VF defects could be detected by SAP^3^.

On the structural side, biological heterogeneity of ONH phenotypes, with or without any neuropathy, can present challenges to clinical decision-making. For instance, the NRR area has been found to normally decline at the rate of 0.28%-0.39% per year^4^. There is no single, specific management guidance for patients with diverse morphology of ONH^5^. For instance, to assess the progression of glaucoma, one of the parameters assessed is the optic cup to optic disc ratio (CDR) which is calculated by comparing the diameter of the “cup” portion of the optic disc with the total diameter of the latter. Yet, while a large CDR may indicate glaucoma or other pathology, deep yet stable (over age) cupping, i.e., a normal physiologically large optic disc cup, can occur due to genetic factors in the absence of any disease or associated clinical covariates (e.g., high intraocular pressure)^6^. It is of great importance that sources of natural variation are rigorously understood thereby controlling for subjectivity in diagnosis.

In the clinic, non-invasive, high-resolution eye imaging platforms such as spectral-domain optical coherence tomography (SD-OCT) provide excellent glaucoma diagnostic performance, especially during early stages of the disease^7,8^. The quantitative and reproducible OCT data provide objective measurements of ONH parameters, NRR area, retinal nerve fiber layer (RNFL), macular thickness, etc., which inform clinicians about structural damage. For instance, Zeiss Cirrus HD-OCT platform uses the clinically invisible but OCT detectable Bruch’s membrane opening (BMO) as the landmark to measure the amount of NRR tissue in the optic nerve. It has been reported that NRR thickness calculation by Cirrus HD-OCT has high reproducibility and glaucoma diagnostic ability, and a low rate of incorrect optic disc margin detection^9–11^. The platform generates a comparative report of a patient's data based on its normative database.

The OCT platform performs circular scans of the eye which, as in many biomedical technologies, are examples of measurements that are recorded or indexed at different directions, say, at given angular positions around a central point. Unlike the analyses of “linear” data points that reside on the real line or Euclidean spaces, directional data requires special and altogether different treatment. For instance, a direction in two-dimensional plane can be represented as a point on the circumference of a unit circle, or simply as an angle, but neither representation is unique, as both depend on the selection of some appropriate “zero-direction” from which to start measuring, as well as the sense of rotation, viz., clockwise or anti-clockwise. The unique properties of circular data—for instance, if one wishes to compare two such scans with a distance measure—are appropriately addressed by the field of circular statistics^12^.

Traditional OCT analysis may involve division of the circle around ONH into 4 fixed quadrants, or 12 clock-hours, to record measurements at these sectors. In this study, we extended such data collection to divide the same circle (of total 360 degrees) into much finer segments of 2 degrees each. Thus, we generated 180 circular data points measuring NRR thickness for each clinical sample (human eye). These rich and evenly spaced high-resolution circular data allow for natural application of functional data analysis (FDA) where the data are not viewed as points but as curves or mathematical functions. Not to be confused with alternate usage of the term “function” (such as in vision), FDA is increasingly popular in biomedical informatics due to the emergence of new monitoring technologies that can record data as curves^13–15^. The high-resolution OCT data for each sample can be modeled as a circular function or curve, with angle and magnitude being the independent and dependent variables, respectively. The circular nature of the OCT NRR data is captured rigorously by the use of Fourier basis functions for their representation as functional or curve data^16^. In this study, we describe a novel method for functional clustering of OCT NRR curves, and apply it for unsupervised identification of NRR phenotypes in healthy Asian Indian eyes. We also address the disparity of representation of Asian Indian eye phenotypes in normative OCT databases.

Progressive and degenerative eye diseases benefit from pre-existing knowledge and cumulative collection and description of normal phenotypes such as NRR may help to identify early and characterize precisely the new phenotypes that emerge over time. While some normative OCT databases do exist, they are generally limited in their size and diversity. Thus, the breakup of their ethnic representation may not reflect the actual epidemiologic distribution of the disease. For instance, only 1% of the popular Cirrus HD-OCT platform’s normative database is of Asian Indian origin^17^, although India contributes to more than 12% of the global cases of both primary open-angle and primary angle-closure glaucomas^18^. Towards this, we generated a new and large high-resolution OCT dataset on NRR phenotypes, along with other clinical covariates, of 3973 healthy eyes as part of a well-established clinical cohort (LVPEI-GLEAMS) at the L.V. Prasad Eye Institute, Hyderabad, India.

The main objectives of the present study are to: (1) generate OCT NRR data in the form of 180 circular measurements of NRR thickness in a given eye, (2) introduce CIFU, a computational pipeline for CIrcular FUnctional data modeling and analysis that is demonstrated using the OCT NRR dataset, and (3) address the disparity of representation of the Asian Indian population in normative OCT databases. In the next section, we describe the clinical cohort and the protocol for data generation as well as the algorithm of CIFU for unsupervised circular functional clustering of the NRR thickness data, followed by meta-clustering to characterize the clustering output using clinical covariates of glaucoma. In the following section, the results of CIFU analysis are described with help of circular visualization. In particular, upon stratification of the samples by age, we identified a healthy NRR phenotype cluster in the age group 40–49 years, and having the highest mean values of cup volume and average CDR among all clusters, with predictive potential for glaucoma. We end with discussion of the CIFU approach and its potential applications to future work.

## Data and methods

### Data

All participants were selected from a population-based study conducted by the L.V. Prasad Eye Institute (LVPEI), Hyderabad, India. It is denoted by LVPEI Glaucoma Epidemiology and Molecular Genetic Study^19^ (LVPEI-GLEAMS). The LVPEI-GLEAMS data protocol was approved by the LVPEI Institutional Ethics Committee (LEC 08131). The data on a total of 3973 healthy eyes of which 1981 right eyes (OD) and 1992 left eyes (OS) were collected from 2222 participants from the southern Indian state of Andhra Pradesh, India. Written informed consent was obtained from all participants to participate in the study, and the ethics and review committee of the LVPEI reviewed and approved the methodology and the study was conducted in strict adherence to the tenets of the Declaration of Helsinki. The inclusion criteria used were age ≥ 40 years, male or female, best-corrected visual acuity of 20/40 or better, spherical equivalent of ± 6 Diopters, good quality stereo optic disc photographs, and no media opacities. The exclusion criteria used were intraocular surgery within the previous 6 months, and any retinal (including macular) or other neurologic diseases that could confound the structural measurements with SD-OCT.

The performance of the OCT layer segmentation algorithms can be affected by poor image quality, leading to erroneous demarcation of the retinal layers and inaccurate measurements. We excluded any OCT scan image sample from our study with poor image quality, signal strength < 6, motion artifacts, blinking artifacts, misidentification of inner and outer retinal layers, and off-center artifacts. Several independent studies, including by some of the authors of the present study, have shown that signal strength reduction is associated with decreased accuracy of nerve fiber layer thickness measurement by OCT, which may be erroneously interpreted as presence of glaucomatous damage on a cross-sectional evaluation or when multiple scans are compared^20–22^. For the Cirrus SD-OCT platform used in the present study, we followed the manufacturer’s definition of scans of adequate quality to be those of signal strength 6 or above (within a range from 0 to 10)^17^.

Healthy eyes were defined by the absence of anterior and posterior pathology. Each digital optic disc photograph was evaluated by three glaucoma specialists independently. The specialists were masked to the other clinical findings and the other imaging outcomes of the subjects. Eyes were excluded from the study in case of any disagreements among the specialists. All participants underwent a comprehensive ophthalmic examination which included detailed medical and systemic history. The means of clinical determination included best-corrected visual acuity measurement, slit-lamp photographs (Topcon, Bauer Drive, Oakland, NJ), Goldmann applanation tonometry (Hagg-Streit AT 900, Hagg-Streit AG, Switzerland), gonioscopy with a Sussman four mirror gonioscope (Volk Optical Inc, Mentor, Ohio, USA), dilated fundus examination, central corneal thickness (CCT) assessment, Humphrey visual fields (HVF) with 24-2 Swedish Interactive threshold algorithm (Carl Zeiss Meditec Inc. Dublin, CA). Visual fields (VF) were considered if false positive, false negative, and fixation losses were less than 20%, and all the stereophotographs of the optic disc had good quality.

In addition, digital optic disc photography and SD-OCT imaging with Cirrus HD-OCT (software version 9.0.0.281; Carl Zeiss Meditec, Dublin, CA, USA) were used. This is a computerized instrument that acquires and analyzes cross-sectional and three-dimensional tomograms of the eye using SD-OCT technology. The instrument’s algorithm automatically identifies the optic disc margin as the termination of Bruch's membrane (BM). BM opening (BMO) is used as the landmark to measure the amount of NRR tissue in the optic nerve. Optic Disc Cube 200 × 200 protocol was used to scan the ONH and peripapillary area through a 6 mm square grid, which consists of 200 horizontal linear B-scans and each composed of 200 A-scans. First, the Cirrus HD-OCT algorithm identifies the center of ONH and then automatically places a calculation circle of 3.46 mm diameter evenly around it. The circular scan starts at an extreme temporal point and moves around the ring in the superior direction, then nasal, then inferior, then back to temporal (TSNIT). The circular measurements are made clockwise for the right eye and counter-clockwise for the left eye. NRR thickness is measured by the amount of neuro-retinal tissue in the optic nerve around the entire edge of the optic disc. Zeiss Cirrus HD-OCT used Bruch's membrane opening–minimum rim width (BMO-MRW) to measure the rim area. The BMO-MRW is the shortest distance from BMO to the retinal internal limiting membrane. The advanced export functionality was used to record the NRR thickness values at 180 points in TSNIT order spaced evenly by 2 degrees (from 2° to 360°) around the circle. We refer to this as our NRR OCT high-resolution circular data. The data were stratified into 3 age groups: (1) 40–49 years, (2) 50–59 years, and (3) 60 years and older.

### Methods

**Figure 1:**
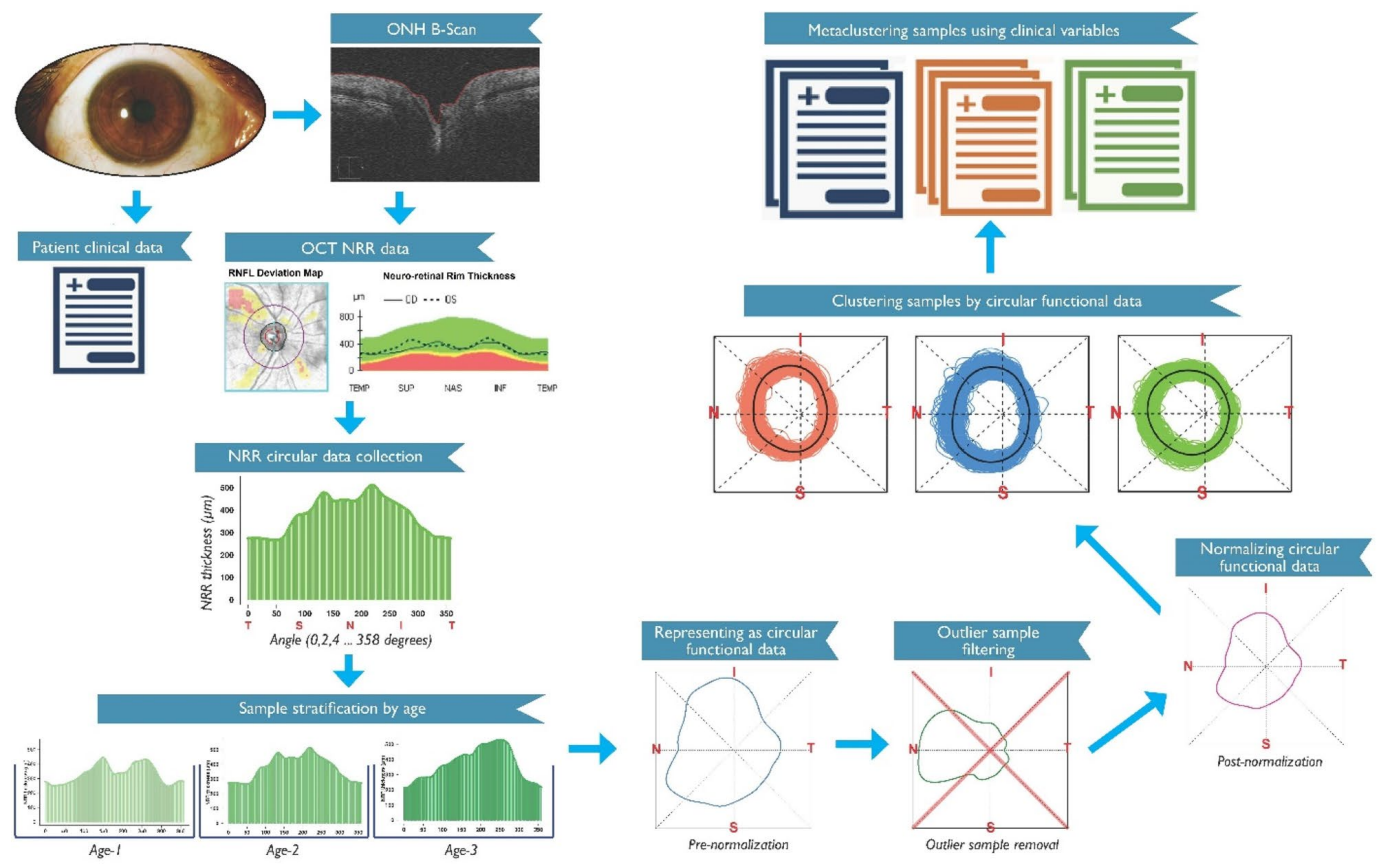
CIFU pipeline gives an illustration of each step including the collection of OCT NRR data and clinical data, representation as and clustering of NRR functional data, and metaclustering of clusters using clinical variables.

We describe CIFU pipeline for circular functional modeling and clustering of OCT NRR data, followed by metaclustering-based clinical characterization of the clusters identified by CIFU. The steps of the CIFU pipeline are graphically illustrated in Fig. 1.

### Circular functional modeling and clustering

First, we introduce a method for clustering OCT NRR data into $K$ homogeneous groups of samples, i.e., eyes. As an unsupervised approach, the clustering is based only on NRR data as input, and not any clinical variables of the samples. While the actual OCT measurements are taken on a discrete grid of angles around the center of ONH, in principle, these finely indexed measurements are assumed to vary continuously around a circular scale ranging from 0 to 360 degrees, and wrapped around. Thus, for statistical modeling, we will refer to the OCT data as a collection of $n$ circular curves ${X}_{1}\left(t\right), {X}_{2}\left(t\right)\dots , {X}_{n}(t),$ where ${X}_{i}(t)$ represents the NRR thickness in the ${i}^{th}$ sample ($i=1, 2, \dots n$) measured at angle $t\in [\mathrm{0,360}]$ and around a common center. The angular indices ${t}_{l}$, where $l=1,\dots 180$, at which the curves are actually measured are aligned for all samples, and spaced 2 degrees apart. Specifically, the $l$-th measurement angle is ${t}_{l}=2(l-1)$.

Our modeling begins with the use of $p$ basis functions for capturing the functional nature of data. If ${\psi }_{1}$, ${\psi }_{2}$,..., ${\psi }_{p}$ are the basis functions with the associated basis expansion coefficients ${\gamma }_{ij}$, where $i=1, 2, \dots n$ and $j=1, 2, \dots p$, then the functional approximation for the ${i}^{th}$ curve at point $t$, ${X}_{i}\left(t\right)$ , is given by

1 $${X}_{i}\left(t\right)\approx \sum_{j=1}^{p}{\gamma }_{ij}{\psi }_{j }\left(t\right)={X}_{i}^{p}(t)$$

Different sources of artifacts could be present in OCT data^23^. As mentioned above, we exclude samples with such artifacts, yet OCT NRR curves with highly unusual shapes might appear rarely. While precise characterization of an outlier curve may be difficult^24^, we used an intuitive criterion for detecting outlier NRR curves using Eq. (2) below, in which the factor 3.5 is based on our exploratory analysis. A given curve ${X}_{i}$ is considered an outlier if there exists a point ${t}_{l}$ for which ${X}_{i}({t}_{l})$ exceeds the following threshold (in terms of mean and sd defined below)

2 $${X}_{out}= \underset{l}{\mathrm{max}}\; {\overline{X} }\left({t}_{l}\right)+3.5 \times \underset{l}{\mathrm{max}} \; sd({X}({t}_{l}))$$

After the outlier curves were removed, to allow for comparison of the curves based on their shapes rather than the magnitude, we normalize each curve ${X}_{i}(t)$ by dividing it by ${\int }_{0}^{360}{X}_{i}\left(t\right)dt$, where the integral is approximated numerically. Note, the normalization step is optional in the CIFU pipeline.

**Figure 2:**
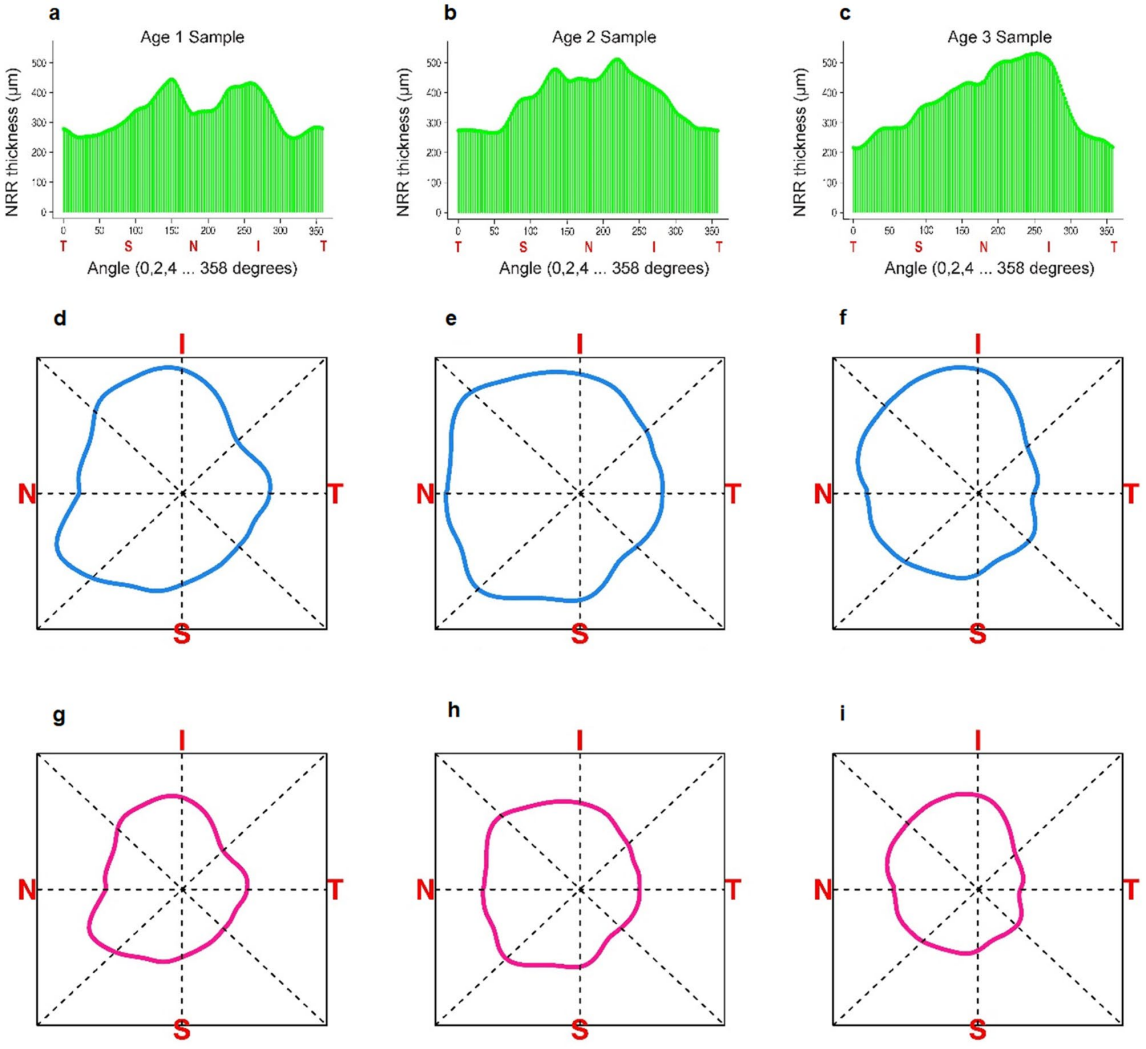
For 3 real samples, selected from each age group (1 to 3 from left to right), the NRR thickness data measured for 180 evenly-spaced points around the circle is shown in the top panel. The corresponding circular functional approximations are shown as pre- and post-normalized NRR curves in the middle and the bottom panels respectively. The direction of the NRR curves is given by TSNIT (clockwise).

As the NRR thickness values are measured radially around a circle (Fig. 2), they are nonnegative and have the natural periodicity of 360 degrees, so that the value corresponding to 5 degrees is the same as that at 365 degrees. After performing the normalization described above, they also contain a unit area on $[$0, 360), and thus possess the properties of a probability density around the circle. Such “circular densities” can be expressed in terms of an infinite series of Fourier coefficients^12^ and then approximated by the first $p$ terms, for a sufficiently large $p$, of the expression in Eq. (1). The idea is similar to approximating a continuous function by a polynomial of high enough degree, except that the Fourier basis also retains the periodicity that is critical to interpretation of the normalized OCT NRR curves.

Our objective is to cluster each of the curves described above into a pre-specified number ($K$) of clusters. Then, we used a discriminative functional mixture (DFM) model given by Bouveyron et al.^23^ in which ${\gamma }_{i}={\left({\gamma }_{i1} , \dots , {\gamma }_{ip}\right)}^{t}$ of curve ${X}_{i}$ follows a finite mixture model of $K$ Gaussian components with density function

3 $$f\left(\gamma \right)= \sum_{k=1}^{K}{\pi }_{k}\varnothing \left(\gamma ;{U{\mu }_{k}},{U}^{t}{\sum }_{k}U +\Xi \right) ,$$

where ${\pi }_{k}\ge 0$ is the mixing proportion of the ${k}^{th}$ component (i.e., the cluster $k$) such that ${\sum }_{k=1}^{K}{\pi }_{k}=1$, and $\varnothing$ is the standard Gaussian density function. Here, $U$ is a $p\times d$ orthogonal matrix mapping the basis coefficients $\gamma$ into the discriminative subspace (of dimension $d<p$) through a linear transformation. Similarly, ${\mu }_{k}$ and ${\Sigma }_{k}$ are the mean vector and covariance matrix (for cluster $k$) of $\gamma$ mapped into the discriminative subspace, and the noise of the above transformation is normally distributed with mean zero and covariance $\Xi$.

The DFM model was fit with an Expectation–Maximization (EM) algorithm implemented in the R package funFEM^24^. EM is a popular iterative method used for optimal fitting of a model and estimation of the model parameters^25^. Following the idea of constraining variance parameters in Fraley and Raftery^26^, the funFEM package allows 12 different choices of DFM models. As an initial step, using different restrictions on the noise covariance matrix $\Xi$, a preliminary model search was run with NRR data, with outlier curves included, over all 12 models and a flexible set of (61) basis functions. Based on the results of the initial run, and after removing the outlier curves as described below, the clustering algorithm was run using a smaller selective set of (21) basis functions.

To avoid model overfitting, we determined the smallest number of basis functions ($p$) that recover the input curves sufficiently well, as determined by the fraction of variation explained ($FVE$) as described below. Let the sample mean and standard deviation of $n$ given curves be respectively

4 $$\overline{X}\left( {t_{l} } \right) = { }\frac{1}{n}\mathop \sum \limits_{i = 1}^{n} X_{i} \left( {t_{l} } \right), \;\;sd(X\left( {t_{l} } \right)) = \{{ }\frac{1}{n-1}\mathop \sum \limits_{i = 1}^{n}( X_{i} \left( {t_{l} } \right)-\overline{X}\left( {t_{l} } \right))^2\}^{\frac{1}{2}},\;\;\;l = 1 , 2 , \ldots 180.$$

Then the total variation (TV) is given by

5 $$TV=\frac{1}{n-1}\sum_{i=1}^{n}\int {\left({X}_{i}\left(t\right)- \overline{X }\left(t\right)\right)}^{2}dt$$

and the fraction of variation explained (FVE) by

6 $$FVE=\frac{TV- \frac{1}{n-1}\sum_{i=1}^{n}\int {\left({X}_{i}^{p}\left(t\right)-{X}_{i}\left(t\right)\right)}^{2} dt}{TV}$$

where the integrals are again approximated numerically using the discrete observations ${X}_{i}\left({t}_{l}\right)$.

We used $FVE$ as the criterion for selecting an optimal number of basis functions ($p$) by identifying the smallest value of $p$ for which $FVE$ exceeds 0.99, i.e., 99% (Supplementary Fig. S1).

Finally, the number of clusters identified by the DFM models for each age group was determined by 3 popular model selection criteria: AIC (Akaike Information Criterion)^27^, BIC (Bayesian Information Criterion)^28^, and ICL (Integrated Complete Likelihood)^29^.

For intuitive visualization of the clustering results, we plotted the curves of every cluster in a distinct color using a circular scale. The mean curve of each cluster, as computed by (4), is included as a bold black curve, which serves as a cluster-specific template.

The R modules of CIFU for fitting the basis functions to OCT data, clustering and visualizing them as circular curves are available from the authors upon request. For each age-group, the de-identified and normalized OCT NRR data for each eye, along with the CIFU-assigned cluster Id, are given in the Supplementary Table S1. For each CIFU-identified cluster, its size, the estimated basis coefficients of its mean circular curve, and its total variation given by the trace of the sample covariance matrix are given in the Supplementary Table S2.

### Comparative clustering analysis

We performed comparative analysis of our circular functional clustering method against three popular non-functional data clustering methods, namely, k-means^30^, Partitioning Around Medoids (PAM^30^) which is similar to kmeans but uses higher dimensional medoids in place of means, and Gaussian mixture model (as implemented in Mclust^26^). Each of these methods were run with the same OCT NRR data *points*, i.e., not as NRR curves, of each age group. Each method was run with a possible choice of fitting $K=2$ through $10$ clusters for each age group. Thus, we ran the 3 alternative clustering methods for 3 age groups for 9 possible values of $K$. The optimal number of clusters identified by each method was determined with the Average Silhouette Width (ASW), while Dunn Index was calculated as a measure of inter-cluster variation. We used the R packages ‘optCluster’^31^ and ‘factoextra’^32^ for clustering, validation and visualization.

### Metaclustering and clinical characterization of clusters

In the metaclustering step, the clusters identified by circular functional data were grouped based on their samples’ similarity in terms of a selected set of clinical variables that are known covariates of glaucoma. A feature selection step was performed simultaneously to detect the covariates that were the most distinctive across the metaclusters. The metaclustering workflow consists of the following steps:

1. In each age group, we performed agglomerative hierarchical clustering of the clusters given by their mean covariate data with complete linkage, while simultaneously doing feature selection to select a sparse set of covariates that are the most distinctive across the metaclusters.
2. We plotted the metaclusters (identified in Step 1) with age group-specific dendrograms. A flat cut of the dendrograms at a common height threshold was used to distinguish the metaclusters in each age group. The metaclusters that correspond across the age groups are shown as subtrees of matched colors.
3. We visualized using contour plots the corresponding metaclusters of each age group to compare the distributions of the selected covariates across the metaclusters as well as the age groups.

In step 1, a set of 9 covariates were used based on their clinical relevance. The R package ‘sparcl’^33^ was used for agglomerative and sparse hierarchical metaclustering in step 2; the feature selection in this package is done by varying the values of its ‘wbound’ parameter from 2 to 5.

## Results

The CIFU pipeline was run with OCT NRR thickness data and clinical assessment data of a normal cohort consisting of 3973 healthy eyes. The steps of the pipeline began with stratification of the OCT and clinical data by age into 3 age groups with (1) 1841, (2) 1351, and (3) 781 samples respectively. The list of clinical variables is summarized in Table 1. An identical sequence of steps of analysis was followed by CIFU within each age group.

**Table 1:** The clinical variables of the study participants in the three age groups.

Clinical variables	Age Group1	Age Group2	Age Group3
Number of eyes, *N* (OD/OS)	1841(917/924)	1351(677/674)	781(387/394)
Age (years)*	43.95 ± 2.83	53.18 ± 2.76	64.63 ± 5.39
Gender, *N* (female/male)	1256/585	755/596	372/409
BCVA LogMAR*	0.01 ± 0.04	0.03 ± 0.08	0.09 ± 0.11
Spherical equivalent (diopter)*	0.02 ± 0.75	0.16 ± 1.05	-0.17 ± 1.3
IOP (mmHg)*	12.58 ± 2.34	12.49 ± 2.41	12.13 ± 2.38
CCT (µm)*	525.7 ± 32.22	524.28 ± 31.96	517.14 ± 31.62
Axial length (mm)*	22.59 ± 0.74	22.63 ± 0.71	22.6 ± 0.8
Family history of Glaucoma, n, (no/yes)	1835/6	1345/6	781/0
Diabetes mellitus, n, (no/yes)	1720/121	1163/188	666/115
Hypertension, n, (no/yes)	1696/145	1083/268	575/206
RIM area (mm^2^)*	1.36 ± 0.22	1.34 ± 0.23	1.33 ± 0.25
Disc area (mm^2^)*	1.96 ± 0.35	1.97 ± 0.35	2.01 ± 0.37
Average CDR*	0.52 ± 0.15	0.53 ± 0.14	0.55 ± 0.14
Average thickness (µm)*	94.36 ± 9.25	92.3 ± 9.57	90.73 ± 9.9
Vertical CDR*	0.49 ± 0.15	0.5 ± 0.13	0.52 ± 0.13
Cup volume (mm^3^)*	0.18 ± 0.16	0.18 ± 0.15	0.2 ± 0.18
Disc diameter (mm)*	1.49 ± 0.15	1.51 ± 0.15	1.53 ± 0.16

The units are given in parentheses. The asterisk (*) denotes that a variable is described as mean ± sd.

*N* number of samples, *OD* oculus dexter, *OS* oculus sinister, *BCVA LogMAR* best corrected visual acuity logarithm of the minimum angle of resolution, *IOP* intraocular pressure, *CCT* central corneal thickness, *CDR* cup-to-disc ratio, *sd* standard deviation.

The 180-point data for each sample (eye) were modeled using $p=11$ Fourier basis functions. We chose $p=11$ since it was the smallest value of $p$ for which $FVE$, as given in Eq. (6), exceeded 99% (Supplementary Fig. S1). The curves were normalized and aligned to a common starting angle of 0 degrees to allow for comparison of their shapes around the center of ONH. Using Eq. (2), the outlier OCT curves were removed: 6 samples from age group 1, 1 from age group 2, and 5 from age group 3. Then, within each age group, the curves were clustered by a discriminative functional mixture (DFM) model as described in Eq. (3). The optimal number of clusters ($K$) for each age group was determined by 3 different well-known criteria: AIC, BIC, and ICL (described in “Methods”). These criteria showed overall strong agreement attesting to optimal model selection as seen in Supplementary Fig. S2. Based on the value of $K$ beyond which no significant gain was noted in these criteria, we determined the number of clusters, for age group 1, 2, and 3 as 7, 8, and 6 respectively. The statistics of each cluster are given in Supplementary Table S2.

**Figure 3:**
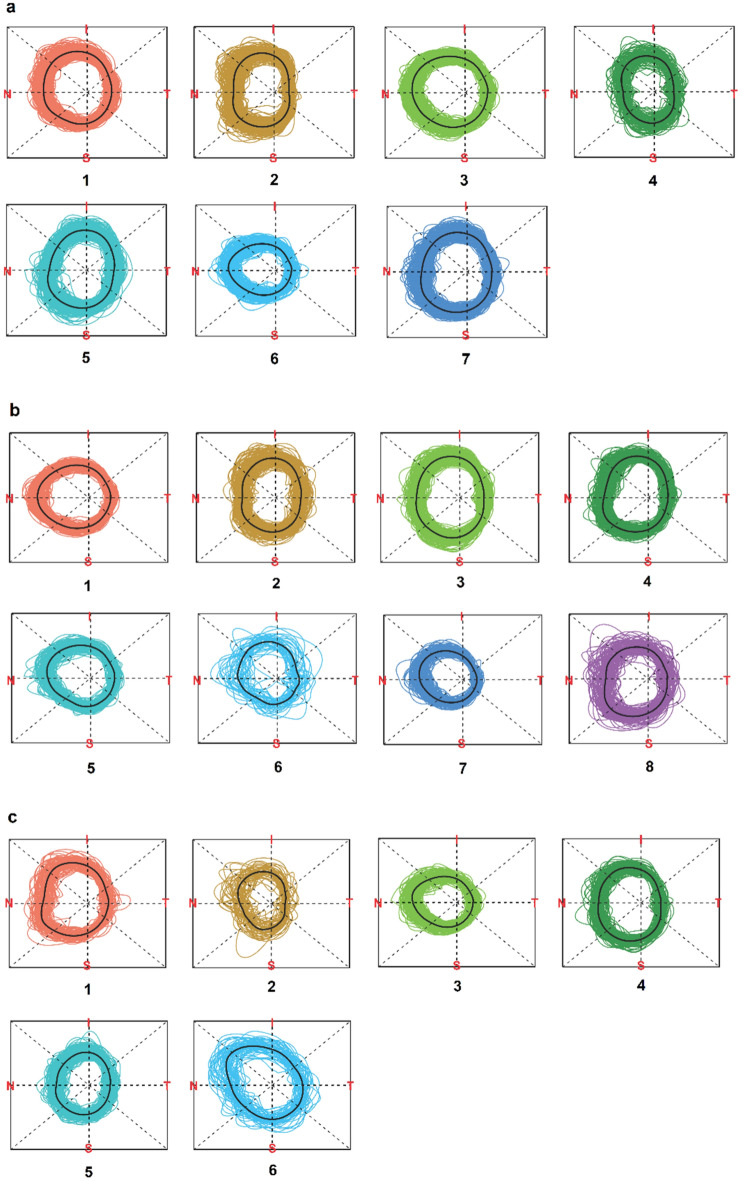
The clusters of the OCT NRR functional data are shown. For each of the age groups (**a**) 1, (**b**) 2, and (**c**) 3, the normalized NRR curves that belong to the same cluster are shown together using a common color. In age groups 1, 2, and 3, CIFU identified 7, 8, and 6 clusters respectively. For each cluster, its mean NRR curve is shown in black. The direction of the NRR curves is given by TSNIT (clockwise).

The results of our circular functional clustering are shown in Fig. 3a–c as a panel of $K$ clusters for each age group. Each cluster $C$ within a panel consists of the circular curves for the samples that belong to $C$ (all shown in a common color specific to $C$) based on the similarity of their functional representation. To gain an intuitive understanding of the 180-point OCT data on NRR phenotypes, we used a visualization of curves as represented on a common circular scale. Unsupervised clustering of the circular functions revealed various NRR patterns in the identified clusters, some of which were distinctive whereas others have subtle differences. Notably, the visualization reveals the unique mean shape (or NRR “template”) of each cluster as shown by a bold black circular curve in each plot of Fig. 3.

The circular curve visualization allows several interesting observations. We note the consistent dip at the temporal (T) region near 0 degrees, which is a characteristic feature shared by the templates of all clusters. This is supported by the well-known ISNT rule^34^ according to which, from the center of ONH, the rim is the thinnest at the temporal (T) region. Interestingly, we also observed various shapes and features in the cluster templates (such as distinctive protrusions, notches, tilts, etc.) that appear as well as vary continuously in different (non-T) regions around the circle. The clustering solution allows us to record the intra-cluster variation which could be used to quantitatively compare the dynamics (say, the rates of focal change) of corresponding clusters across age groups. In this regard, we note that the popular methods of traditional clustering of the same OCT NRR data failed to capture the distinctive shapes and other spatial features of the NRR curves. These methods yielded only 2 clusters of NRR data points (not curves) each in every age group (Supplementary Fig. S3). Further, the traditional clusters (Supplementary Fig. S4) had low inter-cluster structural variation as measured by their small values of Dunn Index for age group (1) kmeans: 0.063, PAM: 0.055, Mclust: 0.043; (2) kmeans: 0.049, PAM: 0.044, Mclust: 0.034; and (3) kmeans: 0.054, PAM: 0.056, Mclust: 0.042.

**Figure 4:**
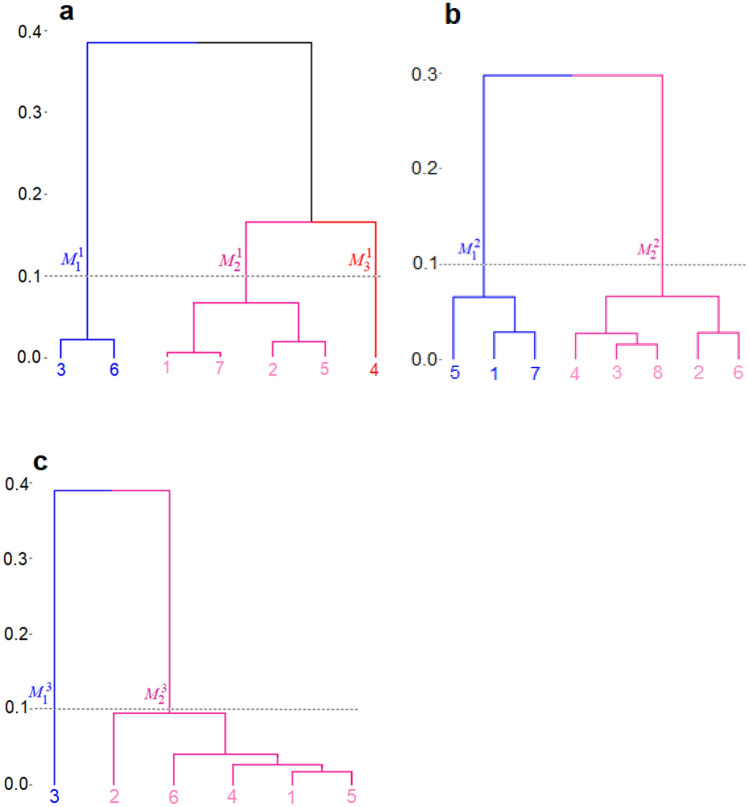
Metaclustering of the clusters was performed using the clinical covariates of the samples in each cluster. The results are shown using dendrograms for age groups (**a**) 1, (**b**) 2, and (**c**) 3. The y-axis shows the distance between metaclusters. The leaves of a dendrogram denote the Id-s of the clusters identified by the previous clustering step. The metaclusters are obtained by a flat cut of each dendrogram at the common height of 0.1, and the labels and subtrees representing them are shown in different colors (blue, pink, and red).

In order to establish a correspondence among the clusters in different age groups as well as to characterize the samples that belong to each cluster, we conducted a metaclustering analysis. In this step, we clustered the clusters based on a set of 9 clinical variables (Table 2) of the samples in each cluster. These are known covariates of glaucoma, and no NRR data from the previous clustering step was used. The results of sparse hierarchical metaclustering are shown in Fig. 4. The dendrograms reveal the similarities among the clusters in terms of their mean sample covariates as well as the counts of metaclusters identified at different levels of each dendrogram. Based on flat cuts of all the dendrograms (at a common height threshold of 0.1), we identified 3 metaclusters {${M}_{1}^{1}, {M}_{2}^{1}, {M}_{3}^{1}$} for age group 1; 2 metaclusters {${M}_{1}^{2}, {M}_{2}^{2}$} for age group 2; and 2 metaclusters {${M}_{1}^{3}, {M}_{2}^{3}$} for age group 3. Notably, all the dendrograms show the metacluster ${M}_{2}^{\cdot }$ (pink subtree) to be more heterogeneous in every age group than the metacluster ${M}_{1}^{\cdot }$ (blue subtree). Among the youngest participants, i.e., in age group 1, the metacluster ${M}_{3}^{1}$ (consisting of the original cluster 4) is distinct from the metacluster ${M}_{2}^{1}$.

**Table 2:** The clinical covariates used for metaclustering in the three age groups.

Metacluster {Clusters}	Age group 1			Age group 2		Age group 3	
	*M*_1_ {3,6}	*M*_2_ {1,2,5,7}	*M*_3_ {4}	*M*_1_ {1,5,7}	*M*_2_ {2,3,4,6,8}	*M*_1_ {3}	*M*_2_ {1,2,4,5,6}
Metacluster *N* (percent)	549 (29.8)	1170 (63.6)	122 (6.6)	547 (40.5)	804 (59.5)	212 (27.1)	569 (72.9)
IOP (mmHg)	12.68 ± 2.31	12.54 ± 2.33	12.54 ± 2.61	12.34 ± 2.31	12.6 ± 2.48	12.12 ± 2.48	12.13 ± 2.34
CCT (µm)	525.93 ± 31.08	526.24 ± 32.86	519.53 ± 30.68	523.06 ± 31.53	525.09 ± 32.23	518.71 ± 33.58	516.56 ± 30.87
Axial length (mm)	22.55 ± 0.75	22.59 ± 0.74	22.7 ± 0.67	22.63 ± 0.69	22.63 ± 0.72	22.8 ± 0.72	22.53 ± 0.82
RIM area (mm^2^)	1.44 ± 0.23	1.32 ± 0.21	1.25 ± 0.19	1.42 ± 0.24	1.29 ± 0.21	1.42 ± 0.25	1.3 ± 0.24
DISC area (mm^2^)	1.86 ± 0.29	1.99 ± 0.35	2.17 ± 0.4	1.89 ± 0.32	2.02 ± 0.36	1.88 ± 0.32	2.06 ± 0.38
DISC Diameter (mm)	1.47 ± 0.13	1.5 ± 0.15	1.59 ± 0.17	1.48 ± 0.14	1.52 ± 0.15	1.48 ± 0.14	1.55 ± 0.16
Vertical CDR	0.45 ± 0.13	0.49 ± 0.15	0.56 ± 0.14	0.47 ± 0.14	0.52 ± 0.13	0.48 ± 0.12	0.53 ± 0.14
**Average CDR**	**0.44 ± 0.13**	**0.54 ± 0.15**	**0.62 ± 0.12**	**0.46 ± 0.14**	**0.57 ± 0.13**	**0.46 ± 0.12**	**0.58 ± 0.14**
**CUP Volume (mm**^**3**^**)**	**0.1 ± 0.1**	**0.2 ± 0.16**	**0.3 ± 0.19**	**0.12 ± 0.12**	**0.22 ± 0.16**	**0.11 ± 0.11**	**0.23 ± 0.19**

The two most significant covariates due to feature selection are shown in bold. The values of each variable in a metacluster are described as mean ± sd. The units are given in parentheses.

*N* the number of samples, *IOP* intraocular pressure, *CCT* central corneal thickness, *CDR* cup-to-disc ratio, *sd* standard deviation.

**Figure 5:**
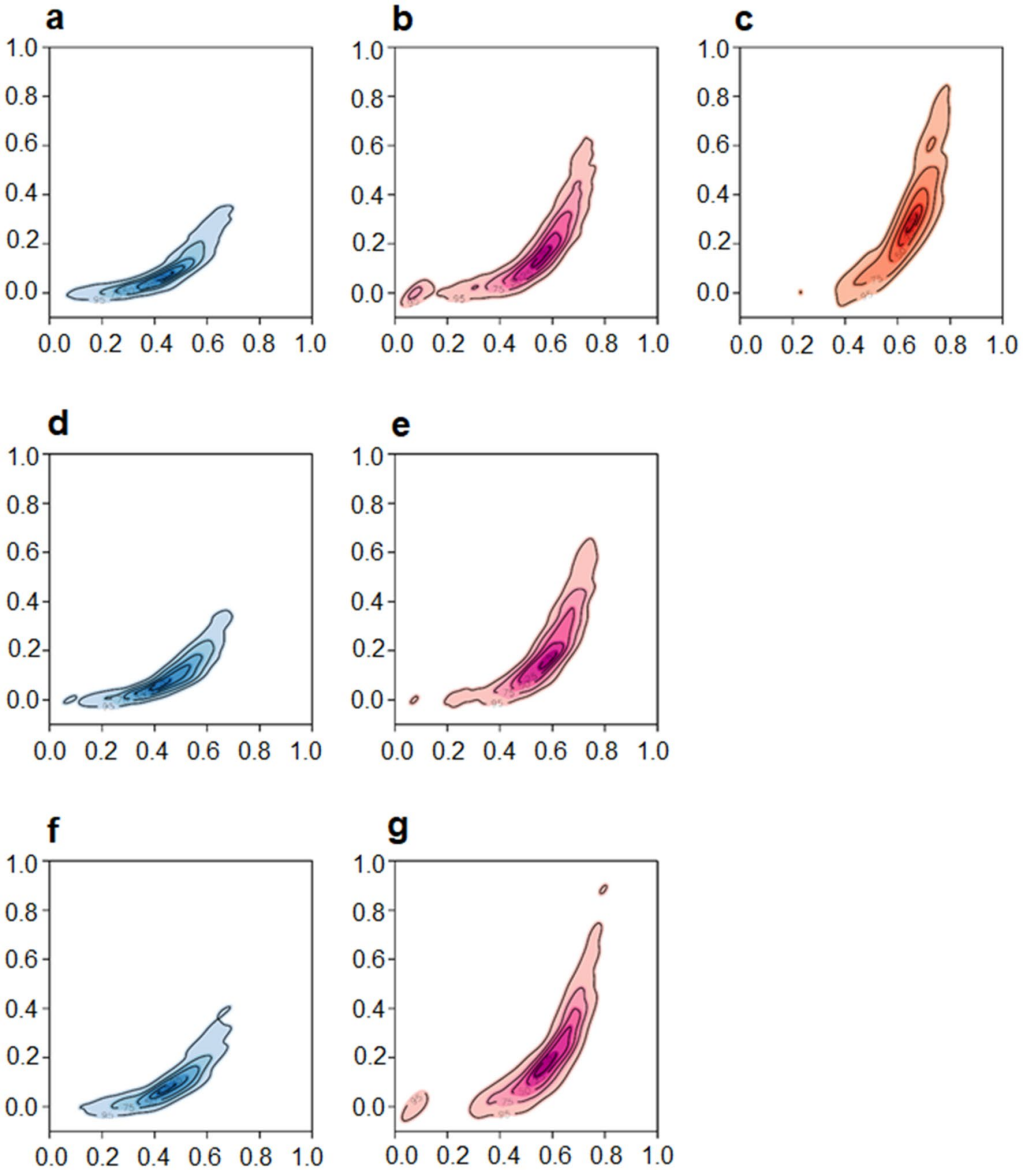
Contour plots of the distributions of clinical covariates optic cup volume (y-axis) and average CDR (x-axis) of the samples belonging to metaclusters of age groups 1: (**a**) ${M}_{1}^{1}$, (**b**) ${M}_{2}^{1}$, (**c**) ${M}_{3}^{1}$; 2: (**d**) ${M}_{1}^{2}$, (**e**) ${M}_{2}^{2}$; and 3: (**f**) ${M}_{1}^{3}$, (**g**) ${M}_{2}^{3}$. The metaclusters that correspond across the 3 age groups are shown in matched colors (blue and pink) while the distinct metacluster (**c**) is shown in red.

A feature selection step, performed along with metaclustering, identified the optic disc cup volume and the average cup-to-disk ratio (CDR) of an eye as the most significant features in terms of the contributions of the different covariates to the metaclustering. These distinctive covariates allow us to register the correspondence of the metaclusters across the different age groups in Fig. 5, which shows the contour plots of the metaclusters in their matched colors. The 3 metaclusters ${M}_{1}^{\cdot }$ shown in blue have the smallest mean values of cup volume and CDR, the 3 metaclusters ${M}_{2}^{\cdot }$ shown in pink have comparatively higher mean values of these covariates, and the single unmatched metacluster ${M}_{3}^{1}$ shown in red (Fig. 5c) has the highest mean values of all metaclusters (Table 2).

It is interesting to consider the unmatched metacluster ${M}_{3}^{1}$ which not only has the highest mean values of the covariates (cup volume and average CDR) but is, in fact, comprised of a single, distinct cluster based on the OCT NRR phenotype data (cluster 4 in Fig. 3a). Here we note that notwithstanding a large value of CDR (especially $>0.5$), cupping by itself is not indicative of glaucoma. In fact, it is known that deep but stable cupping can occur due to hereditary reasons without glaucoma (see “Discussion”). Rather, it is a change in these ONH parameters with age of the participants that is a clinical indicator of glaucoma. Since the samples included in the present study contain only healthy eyes as determined clinically by agreement of multiple glaucoma specialists, the presence of this unmatched cluster only in the youngest age group serves as a signature of healthy NRR phenotype with predictive potential for glaucoma. That is, the corresponding metacluster with such high values of these covariates among the older age groups would have the likelihood of progressing to glaucoma, and thus, is unlikely to be represented in a cohort that consists of healthy eyes only, as we have in the present study.

## Discussion

Unsupervised learning of the heterogeneity of normative ONH phenotypes in a given population can provide a more comprehensive understanding of the diversity of baselines that exist for degenerative neuropathies. Such knowledge is particularly useful in glaucomas for which different ONH parameters play a combined role in early detection. For example, in a non-glaucoma multiethnic cohort of Asian individuals, the inter-eye RNFL profile was found by OCT to be less symmetric in Malays and Indians than that in Chinese eyes^35^. Not only are the structural characteristics of individual eyes known to vary racially, even their rates of change over time could be different across population groups. For instance, the rate of change of BMO-MRW was recorded as –1.82 μm/year and –2.20 μm/year in glaucoma suspect eyes of European and African descents respectively^36^. In another multi-centered normal population study, both age-related decline and between-subject variability in BMO-MRW were observed^37^. Indeed, even the manufacturers of OCT technology noted racial differences in optic disc area, CDR, cup volume, and RNFL thickness when measured using their platform^38^.

The presence of phenotypic heterogeneity makes it less justified to apply common, universal thresholds for clinical determination of glaucomatous damage in different population groups using OCT measurements, particularly in the early stages of the disease when the baselines could have stronger initial effects. To account for the effects of normal variation in ONH parameters, large and racially representative normative databases of healthy eye OCT phenotypes should be created. However, often such collections of healthy samples tend to be small or moderately sized, e.g., the normative database of Cirrus HD-OCT platform included just 284 subjects^38^. In that cohort, Caucasians represented 43%, Chinese 24%, African Americans 18%, Hispanics 12%, and others 6%. The representation of Asian Indians, in contrast, was about 1% of the Cirrus HD-OCT cohort, which does not adequately reflect the 2020 projections about India to become the second in global glaucoma numbers, surpassing Europe^39^. Thus, more than 16 million Indians could be affected by glaucomas, and nearly 1.2 million could be blinded from the disease. Some resources such as the HRT3 Normative Database, while including 104 Indian individuals, did not improve the diagnostic sensitivity or specificity for glaucoma in that group for the potential reasons of limited sample size and intra-racial variation of ocular topography^40^.

In this study, we leveraged the large population-based LVPEI-GLEAMS study to generate new and a relatively large OCT dataset based on nearly 4000 samples from normal Asian Indian participants. In fact, given that the recruitment of all the study participants was from a single geographic region (namely, the state of Andhra Pradesh), the scope of intra-racial variation to affect this dataset is limited. Moreover, the relatively large sample size of the data allowed us to identify a variety of clusters of NRR phenotypes, including the signature (cluster 4) with predictive potential for glaucoma consisting of 6.6% of all samples in the youngest age group (40–49 years). The absence of its corresponding cluster in the older healthy age groups despite their considerable sample sizes (total of 2132 samples of age 50 years and above) leads to a reasonable supposition of its potential pathological progression with increase in age, thereby resulting in lack of subsequent representation in a healthy cohort such as in the present study. Such phenotypic decline is consistent with the findings from a prospective longitudinal study that found the rate of age-related, glaucomatous global (or global percentage) rim area loss to be 3.7 (5.4) times faster as compared to healthy eyes^41^.

Importantly, independent support for the identified signature relies on its clinical characterization in terms of covariates such as cup volume and CDR, which are useful parameters for diagnosis of glaucoma suspects^42^. Despite its normal mean value of intraocular pressure (IOP) as is expected of healthy eyes, the signature cluster has the highest mean values of average CDR and cup volume of all metaclusters across all 3 age groups (Table 2). In a recently published longitudinal study that started from baseline values and was run over a 5-year period, the covariates which had statistically significant increase in glaucomatous progression included CDR and cup volume^43^. We understand that it would perhaps be ideal to follow-up healthy individuals and measure the changes in their clinical covariates as they age in order to classify the ONH phenotypes via supervised learning. The approach of CIFU, in comparison, involves unsupervised learning of different high-resolution phenotypes in age-stratified data and their characterization using key covariates, which is far less time-consuming and yet has the potential to produce a clinically insightful database for diverse populations with high phenotypic heterogeneity.

In addition to its sample size and racial representation, perhaps the most noteworthy feature of the present OCT dataset is its unique high-resolution measurements of NRR thickness around a circle. These 180 samples, collected at every 2 degrees, extend the typical use of such measurements recorded at either 4 quadrants or 12 clock-hours, and even 48 angular positions^44^, to higher-dimensional analysis. As clustering with curves show, the focal variations could be more nuanced than that suggested by a general rule, e.g., ISNT, and a capability to “zoom” into finer angular divisions can reveal further patterns^45^. In low-resolution data, it may be difficult to detect focal changes within the confines of pre-determined inflexible sectors. Moreover, the templates of the clusters could also be compared using known tests in shape analysis^46^. Be it circular data from OCT, or optic phenotypes in general, they seem suitable as candidate applications of circular statistics, and yet, we are unaware of any major previous studies in this regard. Further, the high-resolution also allowed the data to be closely approximated by continuous curves, and thus, specified by the corresponding functional representation. While clustering of circular point data^12,47,48^ as well as clustering of curves^49–51^ and (non-circular) functional data^13,14,23,52–54^ have been addressed by past studies, the present clustering of curves in the form of circular functions is possibly a novel application.

We understand that the present study has certain limitations. As we noted above, a prospective cohort study would be better suited to validate the predictive glaucomatous potential of the identified NRR phenotypic signature. We plan to address this in our future work. While high-resolution data could be accessed from the OCT scans, it is not commonly done by the clinical protocols. We hope that by adding user-friendly interfaces to CIFU, we may be able to promote such data acquisition and analysis, especially since the functional representation is independent of the number of observations per sample. Notably, there are several distinct advantages of our approach which could be built upon further in future studies. The estimated parameters of the fitted functional mixture model could be used to test the similarity of ONH phenotypes in normal versus disease conditions, thus allowing us to characterize any changes with precision and rigor.

After a database of phenotypic parameters is developed, known measures of shapes and distances between curves could be used for objective clinical classification of new OCT samples. Applied to longitudinal analyses, our high-resolution modeling could identify intermediate, or previously uncharacterized, stages of disease progression, especially by focusing on variations within fine angular sections. Focused analysis of angular sections of OCT NRR and RNFL data have revealed interesting differences between healthy and glaucoma subjects, and we plan to apply CIFU for mining locally distinctive features in higher resolution^55^. Indeed, straightforward extensions are feasible for similar circular data such as RNFL phenotypes and other optic neuropathies as well as related eye imaging platforms, e.g., OCT-Angiography (OCTA). As we have demonstrated for other biomedical platforms^51,56–59^, the new pipeline CIFU could be enhanced incrementally with different functionalities, say, to increase computational efficiency or capture the perspective of the clinical experts. The circular curve visualization introduced in the present study may lead to a more user-friendly tool for clinical purposes as we plan to make it interactive, with advanced capabilities to jointly handle data and metadata, in our future work.

## Supplementary Information

Supplementary Information 1.

Supplementary Information 2.

Supplementary Information 3.

## References

[1] *Global Initiative for the Elimination of Avoidable Blindness: Action Plan 2006–2011*. (World Health Organization, 2007). https://apps.who.int/iris/handle/10665/43754.

[2] Tham Y-C (2014). Global prevalence of glaucoma and projections of glaucoma burden through 2040: A systematic review and meta-analysis. Ophthalmology.

[3] Kerrigan-Baumrind LA, Quigley HA, Pease ME, Kerrigan DF, Mitchell RS (2000). Number of ganglion cells in glaucoma eyes compared with threshold visual field tests in the same persons. Investig. Ophthalmol. Vis. Sci..

[4] Garway-Heath D, Wollstein G, Hitchings R (1997). Aging changes of the optic nerve head in relation to open angle glaucoma. Br. J. Ophthalmol..

[5] Prata TS (2014). Eyes with large disc cupping and normal intraocular pressure: using optical coherence tomography to discriminate those with and without glaucoma. Med. Hypothesis Discov. Innov. Ophthalmol. J..

[6] Kwon Y, Fingert J, Kuehm M, Alward W (2009). Primary open-angle glaucoma. N. Engl. J. Med..

[7] Leung CKS (2010). Retinal nerve fiber layer imaging with spectral-domain optical coherence tomography: Analysis of the retinal nerve fiber layer map for glaucoma detection. Ophthalmology.

[8] Leung CKS (2010). Retinal nerve fiber layer imaging with spectral-domain optical coherence tomography: Pattern of RNFL defects in glaucoma. Ophthalmology.

[9] Mwanza JC (2010). Reproducibility of peripapillary retinal nerve fiber layer thickness and optic nerve head parameters measured with cirrus HD-OCT in glaucomatous eyes. Investig. Ophthalmol. Vis. Sci..

[10] Mwanza JC, Oakley JD, Budenz DL, Anderson DR (2011). Ability of cirrus HD-OCT optic nerve head parameters to discriminate normal from glaucomatous eyes. Ophthalmology.

[11] Sung KR, Na JH, Lee Y (2012). Glaucoma diagnostic capabilities of optic nerve head parameters as determined by cirrus HD optical coherence tomography. J. Glaucoma.

[12] Jammalamadaka SR, SenGupta A (2001). Topics in Circular Statistics.

[13] Ieva F, Paganoni AM, Davide P, Valeria V (2012). Multivariate functional clustering for the morphological analysis of electrocardiograph curves. Appl. Stat..

[14] Jacques J, Preda C (2013). Funclust: A curves clustering method using functional random variables density approximation. Neurocomputing.

[15] Wang J-L, Chiou J-M, Müller H-G (2016). Functional data analysis. Annu. Rev. Stat. Appl..

[16] Jammalamadaka SR, Sarma Y, Matusita K (1993). Circular regression. Statistical Sciences and Data Analysis.

[17] Carl Zeiss Meditec Inc (2015). Cirrus HD-OCT User Manual.

[18] Thylefors B, Négrel A (1994). The global impact of glaucoma. Bull. World Health Organ.

[19] Addepalli UK (2013). LV Prasad Eye Institute Glaucoma Epidemiology and Molecular Genetic Study (LVPEI-GLEAMS). Report 1: study design and research methodology. Ophthalmic Epidemiol..

[20] Rao H (2014). Effect of scan quality on diagnostic accuracy of spectral-domain optical coherence tomography in glaucoma. Am. J. Ophthalmol..

[21] Samarawickrama C (2010). Influence of OCT signal strength on macular, optic nerve head, and retinal nerve fiber layer parameters. Invest. Ophthalmol. Vis. Sci..

[22] Wu Z, Huang J, Dustin L, Sadda S (2009). Signal strength is an important determinant of accuracy of nerve fiber layer thickness measurement by optical coherence tomography. J. Glaucoma.

[23] Bouveyron C, Côme E, Jacques J (2015). The discriminative functional mixture model for a comparative analysis of bike sharing systems. Ann. Appl. Stat..

[24] CRAN—Package funFEM. https://cran.r-project.org/web/packages/funFEM/index.html.

[25] McLachlan GJ, Krishnan T (2008). The EM Algorithm and Extensions. Wiley Series in Probability and Statistics.

[26] Fraley C, Raftery AE (1999). MCLUST: Software for model-based cluster analysis. J. Classif..

[27] Akaike H (1974). A new look at the statistical model identification. IEEE Trans. Autom. Control.

[28] Biernacki C, Celeux G, Govaert G (2000). Assessing a mixture model for clustering with the integrated completed likelihood. IEEE Trans. Pattern Anal. Mach. Intell..

[29] Manchester L, Blanchard W (1996). When is a curve an outlier? An account of a tricky problem. Can. J. Stat..

[30] Kaufman L, Rousseeuw PJ (2005). Finding Groups in Data: An Introduction to Cluster Analysis.

[31] Sekula M, Datta S, Datta S (2017). optCluster: An R package for determining the optimal clustering algorithm. Bioinformation.

[32] CRAN—Package factoextra. https://cran.r-project.org/web/packages/factoextra/index.html.

[33] CRAN—Package sparcl. https://cran.r-project.org/web/packages/sparcl/index.html.

[34] Jonas JB, Gusek GC, Naumann GO (1988). Optic disc, cup and neuroretinal rim size, configuration and correlations in normal eyes. Invest. Ophthalmol. Vis. Sci..

[35] Tao Y (2020). Profile of retinal nerve fibre layer symmetry in a multiethnic Asian population: The Singapore Epidemiology of Eye Diseases study. Br. J. Ophthalmol..

[36] Bowd C (2018). Racial differences in rate of change of spectral domain OCT-measured minimum rim width and retinal nerve fiber layer thickness. Am. J. Ophthalmol..

[37] Chauhan BC (2015). Bruch’s membrane opening-minimum rim width and retinal nerve fibre layer thickness in a normal white population. A Multi-centre study. Ophthalmology.

[38] Knight OJ, Girkin CA, Budenz DL, Durbin MK, Feuer WJ (2012). Effect of race, age, and axial length on optic nerve head parameters and retinal nerve fiber layer thickness measured by cirrus HD-OCT. Arch. Ophthalmol..

[39] Quigley H, Broman AT (2006). The number of people with glaucoma worldwide in 2010 and 2020. Br. J. Ophthalmol..

[40] Rao HL, Babu GJ, Sekhar GC (2010). Comparison of the diagnostic capability of the Heidelberg retina tomographs 2 and 3 for glaucoma in the Indian population. Ophthalmology.

[41] Hammel N (2016). Rate and pattern of rim area loss in healthy and progressing glaucoma eyes. Ophthalmology.

[42] Garway-Heath DF, Ruben ST, Viswanathan A, Hitchings RA (1998). Vertical cup/disc ratio in relation to optic disc size: Its value in the assessment of the glaucoma suspect. Br. J. Ophthalmol..

[43] Siesky B (2020). Baseline structural characteristics of the optic nerve head and retinal nerve fiber layer are associated with progressive visual field loss in patients with open-angle glaucoma. PLoS ONE.

[44] Reis ASC (2012). Influence of clinically invisible, but optical coherence tomography detected, optic disc margin anatomy on neuroretinal rim evaluation. Invest. Ophthalmol. Vis. Sci..

[45] Garway-Heath DF, Hitchings RA (1998). Quantitative evaluation of the optic nerve head in early glaucoma. Br. J. Ophthalmol..

[46] Dryden IL, Mardia KV (2016). Statistical Shape Analysis: With Applications in R. Wiley Series in Probability and Statistics.

[47] Abraham C, Molinari N, Servien R (2013). Unsupervised clustering of multivariate circular data. Stat. Med..

[48] Rodríguez, C. E., Núñez-Antonio, G. & Escarela, G. A Bayesian mixture model for clustering circular data. *Comput. Stat. Data Anal.* **143**, 106842 (2020).

[49] Gaffney, S. & Smyth, P. Joint probabilistic curve clustering and alignment. In *Proceedings of the 17th International Conference on Neural Information Processing Systems* 473–480 (2004).

[50] Heard NA, Holmes CC, Stephens DA (2006). A quantitative study of gene regulation involved in the immune response of Anopheline mosquitoes: An application of Bayesian hierarchical clustering of curves. J. Am. Stat. Assoc..

[51] Ray S, Pyne S (2012). A computational framework to emulate the human perspective in flow cytometric data analysis. PLoS ONE.

[52] James GM, Sugar CA (2003). Clustering for sparsely sampled functional data. J. Am. Stat. Assoc..

[53] Jones PN, McLachlan GJ (1992). Fitting finite mixture models in a regression context. Aust. J. Stat..

[54] Ray S, Mallick B (2006). Functional clustering by Bayesian wavelet methods. J. R. Stat. Soc. Ser. B.

[55] Hwang YH, Kim YY (2012). Glaucoma diagnostic ability of quadrant and clock-hour neuroretinal rim assessment using cirrus HD optical coherence tomography. Invest. Ophthalmol. Vis. Sci..

[56] Pyne S (2009). Automated high-dimensional flow cytometric data analysis. PNAS.

[57] Ho H, Pyne S, Lin T (2012). Maximum likelihood inference for mixtures of skew Student-t-normal distributions through practical EM-type algorithms. Stat. Comput..

[58] Pyne S (2014). Joint modeling and registration of cell populations in cohorts of high-dimensional flowcytometric data. PLoS ONE.

[59] Qi Y (2020). High-speed automaticcharacterization of rare events in flow cytometric data. PLoS ONE.

