# Supplementary material for: Circular functional analysis of OCT data for precise identification of structural phenotypes in the eye
Source: Sci Rep. 2021 Dec 2;11:23336. doi: 10.1038/s41598-021-02025-4 (PMC8639843; doi:10.1038/s41598-021-02025-4)

**Title:** Circular functional analysis of OCT data for precise identification of structural phenotypes in the eye

**Authors:**

Md. Hasnat Ali^1,2^, Brian Wainwright^3^, Alexander Petersen^3,4^, Ganesh B. Jonnadula^1^, Meghana Aruru^5^, Harsha L. Rao^6,7^, M. B. Srinivas^2^, S. Rao Jammalamadaka^3^, Sirisha Senthil^1^, and Saumyadipta Pyne^5,8,^*

**Author Affiliations:**

^1^ L. V. Prasad Eye Institute, Hyderabad, Telangana, India.

^2^ BITS Pilani, Hyderabad Campus, Hyderabad, Telangana, India.

^3^ Department of Statistics and Applied Probability, University of California, Santa Barbara, CA, USA.

^4^ Department of Statistics, Brigham Young University, Provo, UT, USA.

^5^ Health Analytics Network, Pittsburgh, PA, USA.

^6^ Narayana Nethralaya Eye Hospital, Bengaluru, Karnataka, India.

^7^ University Eye Clinic Maastricht, Maastricht University Medical Center, Maastricht, the Netherlands.

^8^ Public Health Dynamics Laboratory, and Department of Biostatistics, Graduate School of Public Health, University of Pittsburgh, Pittsburgh, PA, USA.

***Corresponding Authors:**

Md. Hasnat Ali, email: hasali4u@gmail.com

Saumyadipta Pyne, email: pyne.saum@gmail.com

**Supplementary Figures**

**Supplementary Figure S1:** The fraction of variation explained ($FVE$) by models with different choices of the number ($p$) of basis functions used for the functional representation of OCT data.


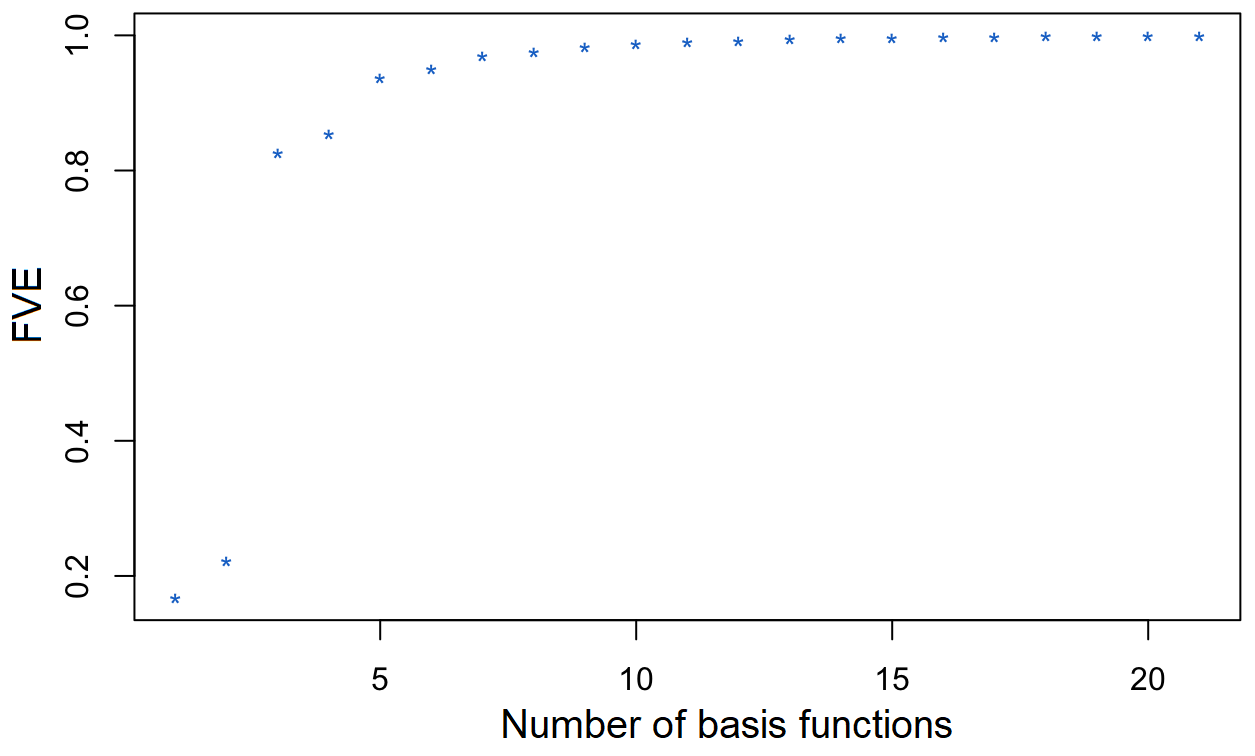


**Supplementary Figure S2:** The values of model selection criteria AIC, BIC, and ICL corresponding to fitting of a DFM model of $K$ clusters to OCT NRR samples of age group 1 shown in (a), 2 in (b) and 3 in (c). The optimal DFM models for the age groups 1, 2 and 3 were selected for $K$=7, 8 and 6 respectively, beyond which no significant gain was noted.


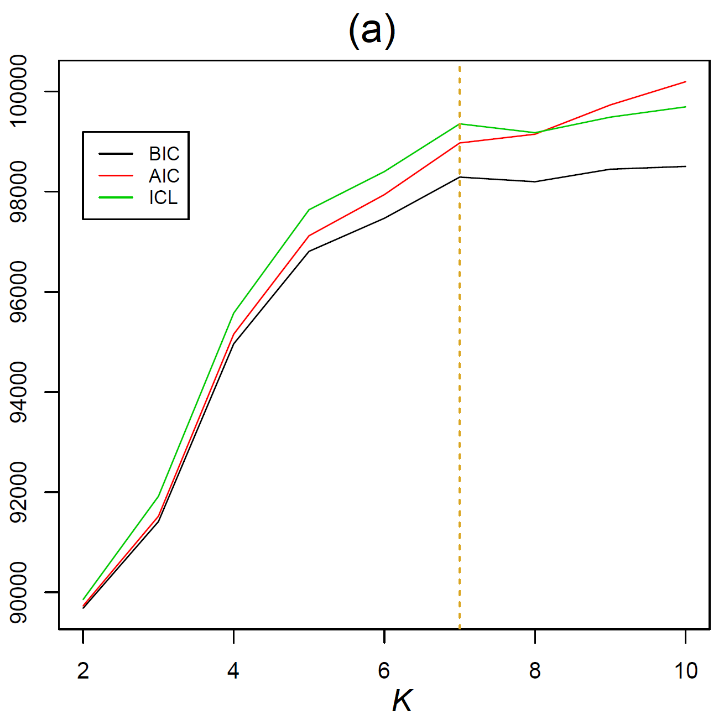

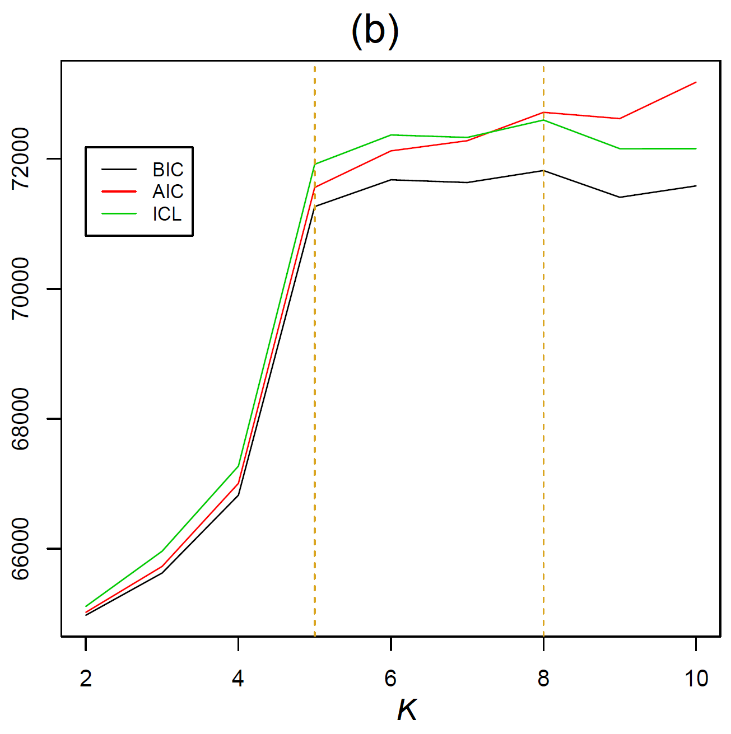


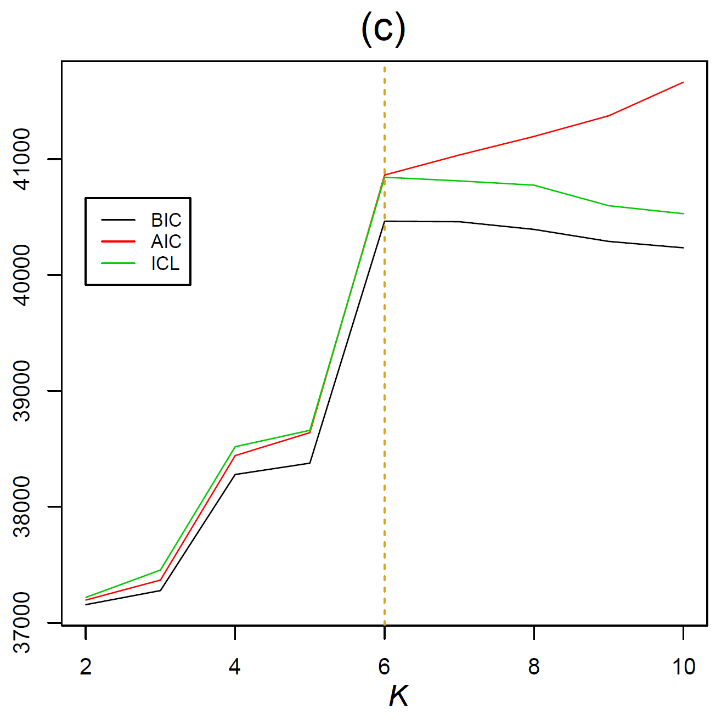


**Supplementary Figure S3:** Non-functional clustering of the normalized OCT NRR data was conducted using 3 popular methods: k-means, PAM, and Gaussian mixture model by Mclust. For age groups (a) 1, (b) 2, and (c) 3, the Average Silhouette Width (ASW in y-axis) for different choices of the number of clusters (*K* in x-axis) is shown. ASW is maximized for *K*=2, thus giving an optimal number of 2 clusters for all 3 clustering methods and for all 3 age groups.


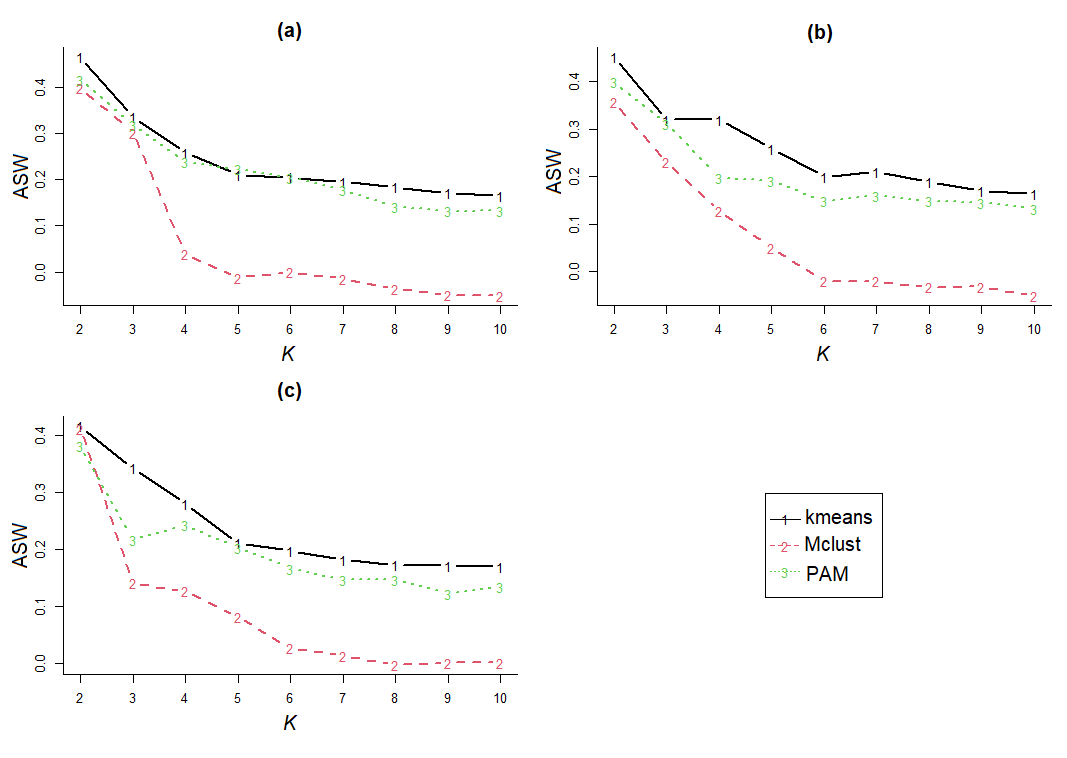


**Supplementary Figure S4:** Non-functional traditional clustering of the normalized OCT NRR data was conducted using 3 popular methods: k-means (a)-(c), PAM (d)-(f), and Gaussian mixture model by Mclust (g)-(i). The age groups are noted on top of each plot. Based on the Average Silhouette Width for each method and each age, the optimal number of clusters is 2. The samples belonging to the 2 clusters are shown in each plot in different colors. Given the high-dimensionality of the data, we used as axes the first two principal components as to visualize the clustering results.


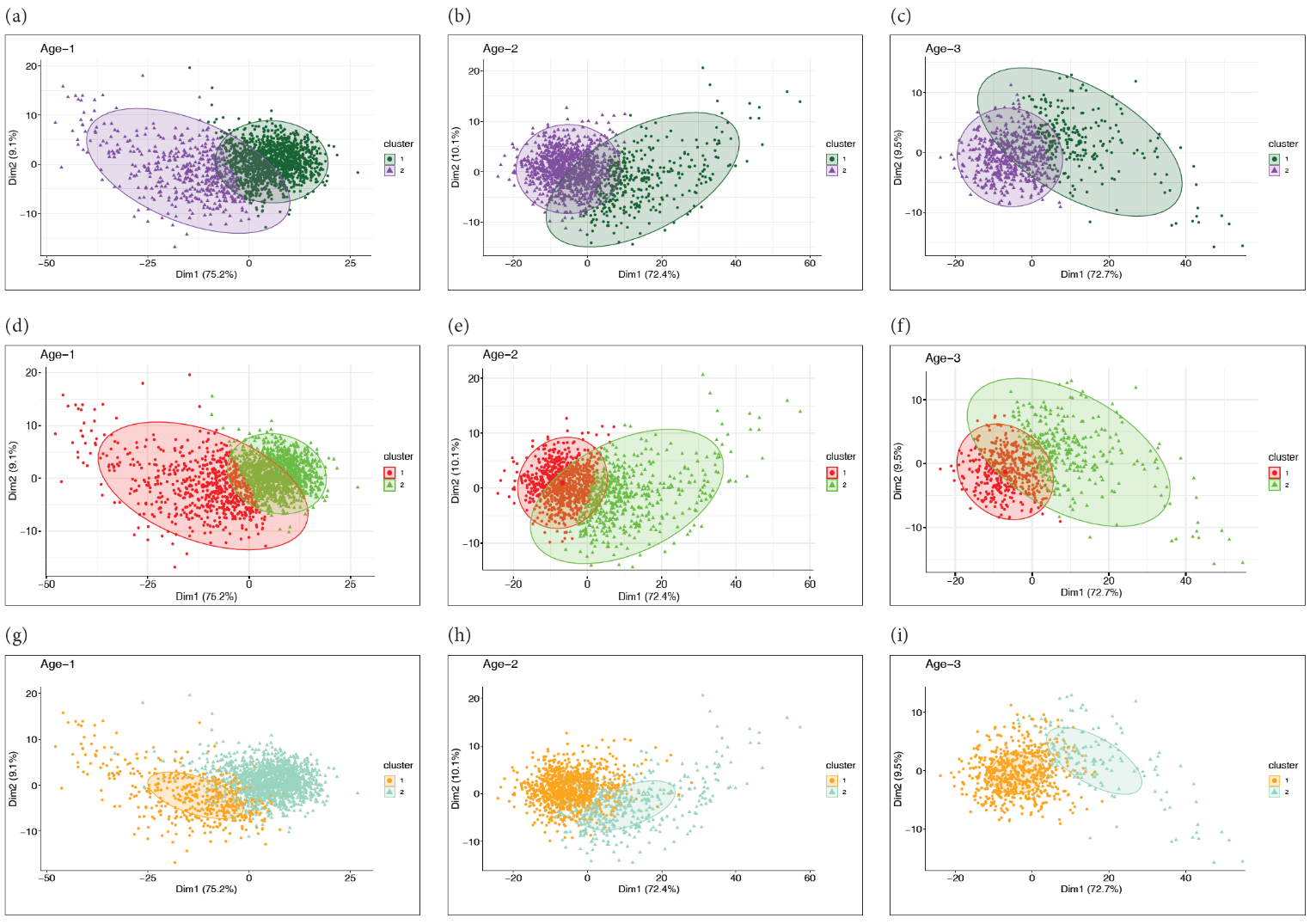

Supplement: Supplementary file 1 — Supplementary Information 1. [file 41598_2021_2025_MOESM1_ESM.docx]
